# Unlocking the Potential of Population Pharmacokinetic Models of Adalimumab in Patients with Crohn’s Disease

**DOI:** 10.3390/pharmaceutics18070788

**Published:** 2026-06-27

**Authors:** Marija Jovanović, Valentina Topić Vučenović, Maša Roganović, Gordana Pavlović, Đorđe Kralj, Srđan Marković, Petar Svorcan, Katarina Vučićević

**Affiliations:** 1 Department of Pharmacokinetics and Clinical Pharmacy, Faculty of Pharmacy, University of Belgrade, 11221 Belgrade, Serbia; 2 Department of Pharmacokinetics and Clinical Pharmacy, Faculty of Medicine, University of Banja Luka, 78000 Banja Luka, Bosnia and Herzegovina; 3 Internal Medicine Clinic, University Clinical Hospital Center “Dr Dragiša Mišović—Dedinje”, 11000 Belgrade, Serbia; 4 Department of Gastroenterology and Hepatology, University Hospital Medical Center “Zvezdara”, 11000 Belgrade, Serbia; 5 Faculty of Medicine, University of Belgrade, 11000 Belgrade, Serbia

**Keywords:** adalimumab, NONMEM, predictive performance, population pharmacokinetic model

## Abstract

**Background/Objectives:** Adalimumab (ADM) is a recombinant, fully human monoclonal antibody that exhibits pronounced inter- and intra-individual pharmacokinetic variability attributed to several factors. This study aims to externally evaluate the published ADM population pharmacokinetic models and their potential use in clinical practice, as well as to develop novel population pharmacokinetic model. **Methods:** Literature search was conducted using PubMed to identify ADM population pharmacokinetic models. Data from 195 patients with Crohn’s disease treated at the University Medical Center “Zvezdara”, Serbia, were used for the external evaluation of previously published models. In addition, the development of the new population pharmacokinetic model incorporated informative priors derived from the best-performing published model. Nonlinear mixed-effects modeling was performed in NONMEM^®^ (versions 7.6) for both prediction- and simulation-based diagnostics of existing models, as well as for the development of a new model. **Results:** Eight published pharmacokinetic models of ADM were included in the external evaluation. Although none of the models satisfied both population-level and normalized prediction distribution error (NPDE) diagnostic criteria, individual-level performance was acceptable: median prediction errors (MDPEs) were within ±20% across all studies, and median absolute prediction errors (MDAPEs) were below 30% in most cases (7 of 8 studies). The best-performing model was identified and implemented as a priori information in subsequent model development. A one-compartment model using with first-order absorption and elimination best described the data. The apparent clearance (CL/F) was estimated at 0.334 L/day, while informative priors were used for V/F and the effect of anti-drug antibodies (ADAs) on CL/F. Covariate analysis on CL/F identified C-reactive protein (CRP) and dosing regimen as statistically significant predictors (*p* < 0.01). **Conclusions:** The previous pharmacokinetic models of ADM exhibited suboptimal performance in population-level metrics and simulation-based diagnostics, while individual-level metrics showed substantial improvement. The newly developed model of ADM highlights associations among immunogenicity, drug pharmacokinetics, and inflammatory burden.

## 1. Introduction

Adalimumab (ADM) is a recombinant, fully human IgG1 monoclonal antibody that binds to and inhibits tumor necrosis factor-α (TNF-α), thereby reducing inflammatory processes [1,2]. According to the European Summary of Product Characteristics (SPC), this drug is indicated for the treatment of several inflammatory diseases, including treatment of adults with moderately-to-severely active Crohn’s disease (CD) or ulcerative colitis (UC) [3]. In CD patients, the recommended induction dosing regimen is 80 mg at week 0 followed by 40 mg at week 2. In patients requiring a more rapid clinical response, an intensified induction regimen of 160 mg at week 0 followed by 80 mg at week 2 is recommended. Following induction, the standard maintenance regimen is 40 mg administered subcutaneously every other week [3]. Despite its efficacy, ADM therapy is associated with secondary loss of response in a substantial number of patients (up to 20% of patients), leading to dose intensification or discontinuation [3,4]. In some patients, increasing the dosing frequency to weekly administration or increasing the dose to 80 mg every other week may provide clinical benefit [3,5].

Various patient-, disease-, and treatment-related factors may interfere with the clearance (CL) of therapeutic monoclonal antibodies, resulting in altered serum concentrations, and consequently clinical response. ADM exhibits pronounced inter- and intra-individual pharmacokinetic variability that has been attributed to several factors, such as sex, body weight, albumin (ALB) level, systemic inflammation, and the formation of anti-adalimumab antibodies (ADAs) [6,7,8]. Thus, therapeutic drug monitoring (TDM), including measurement of ADM concentrations and ADA, plays a pivotal role in therapy optimization. It is typically reactive during the maintenance phase to optimize ADM dosing [9]; however, a proactive approach may also be considered [10]. Improved understanding of the pharmacokinetic variability of ADM, together with factors influencing drug exposure and therapeutic response, may contribute to a more effective implementation of TDM in clinical practice. More importantly, predicting suboptimal pharmacokinetic profiles in certain patients may be essential to mitigate the adverse consequences of insufficient drug exposure. In this context, model-informed precision dosing (MIPD) bridges pharmacometric modeling with clinical practice through the application of Bayesian methodology and population pharmacokinetic models to enable personalized dosing strategies for biologic agents [8,11].

Although several population pharmacokinetic models of ADM have been developed in IBD patients, their suitability for dose individualization may vary considerably across different patient populations. For instance, these models describe distinct sources of pharmacokinetic variability, reflecting the specific characteristics of the underlying patient population used for model development [1]. Consequently, their clinical utility in real-world clinical settings requires external validation in an independent cohort of patients. Furthermore, careful consideration should be given to the population used in model development when selecting a model candidate, as these may not always be representative of the target population [11,12]. In addition, differences in covariate availability, sampling design, and analytical approaches may further limit the direct implementation of published models in routine clinical data. Therefore, while previously published models provide an important reference, their direct applicability to our dataset was not always feasible in a straightforward manner. Accordingly, external evaluation was necessary to assess their predictive performance in our cohort and to determine whether they could reliably support MIPD. In cases where model implementation was limited or performance was suboptimal, the development of a model tailored to the target population may be warranted. Thus, the aim of this study was to evaluate the applicability of existing pharmacokinetic models of ADM in our patient population and to explore their potential use in clinical practice within an MIPD framework. Available population models obtained from the literature describing the pharmacokinetic of ADM in patients with IBD were externally validated. Specifically, we assessed their predictive performance, as well as their utility in simulation-based applications. Moreover, a novel population pharmacokinetic model of ADM was developed, informed by the results of the external evaluation, with the objective of better capturing the variability observed in the target population and supporting subsequent individualized therapeutic optimization.

## 2. Materials and Methods

### 2.1. Literature Search

A systematic search of the PubMed database was undertaken to identify original publications reporting population pharmacokinetic models of ADM in patients with IBD, covering studies published up to February 2026 and limited to the English language. The population studies were searched using the following terms: (Adalimumab) AND (pharmacokinetic model OR population pharmacokinetics OR population pharmacokinetic model* OR nonlinear mixed-effects model* OR NONMEM OR pharmacometric) AND (Inflammatory Bowel Diseases OR Crohn Disease OR Crohn’s Disease OR ulcerative colitis). Moreover, the reference lists of the relevant articles were searched for additional literature. The models were excluded if (1) the study did not describe a population pharmacokinetic model of ADM developed or adapted (by a nonlinear mixed-effects modeling approach) for use in IBD patients; (2) model parameters were not available for code reconstruction and external evaluation; (3) the publications were reviews articles; (4) the models were not published in peer-reviewed scientific journals; or (5) the models were developed on a population unrepresentative for our population (e.g., pediatric patients). The following information was extracted from the selected articles: patient characteristics, software used, structural pharmacokinetic model, typical parameter estimates, inter-individual variability (IIV), inter-occasion variability (IOV), residual variability (RV), and covariates identified as significantly influencing apparent clearance (CL/F) and/or apparent volume of distribution (V/F).

### 2.2. Patients

The study included adult patients diagnosed with CD at the University Medical Center “Zvezdara”, Belgrade, Serbia, and treated with ADM, who underwent TDM during either the induction or maintenance phase of treatment. The study protocol was approved by the institutional Ethics Committee (No. IRB00009457; 5 October 2022). The study was designed and conducted in accordance with the ethical principles outlined in the Declaration of Helsinki. ADM (Humira^®^, 40 mg solution for injection in pre-filled pen, AbbVie Biotechnology GmbH, Ludwigshafen, Germany) was administered subcutaneously, according to the recommended induction and maintenance regimens outlined in the SPC [3], with dosing optimization implemented when clinically indicated.

Alongside ADM therapy characteristics (dosing regimen), TDM (date and time of blood sampling and measured serum trough concentrations with median time after dose of 6.75 or 13.75 days), co-therapy, demographic (age, sex, body weight), clinical (disease type) and available laboratory data were recorded. Laboratory data included hemoglobin, hematocrit, erythrocytes, erythrocyte sedimentation rate (ESR), iron, ferritin, platelets, leukocytes, ALB, C-reactive protein (CRP) and fecal calprotectin (FCP). Levels of ADA were categorized as present or absent. The required data were extracted from the patients’ medical records. Patients were excluded from the analysis if ADM dosing and TDM data were missing.

ADM and ADA concentration measurements were performed using commercially available R-Biopharm^®^ ELISA tests on a Dynex DS2 analyzer (Dynex Technologies, Chantilly, VA, USA) in the biochemical laboratory of University Hospital Medical Center “Zvezdara”, Belgrade, Serbia.

### 2.3. External Evaluation of Previous Models

External evaluation was performed using NONMEM^®^ version 7.6 (ICON Development Solutions, Ellicott City, MD, USA) [13]. Published population pharmacokinetic models were reconstructed based on the parameters and equations reported in the original publications. Population (PRED) and individual (IPRED) predicted ADM concentrations were generated using the reconstructed models, which were executed with zero iterations (MAXEVAL = 0). Observations with ADM concentrations below the lower limit of quantification (BLQ) or subset of ADM concentrations qualitatively reported as exceeding the therapeutic range (>12 mg/L) were censored for the purpose of external evaluation of the previous models. For missing continuous covariates, the patient-specific median was imputed; if unavailable, the overall population median was used. Missing values for categorical covariates were imputed as zero. Model predictive performance was evaluated using both prediction-based and simulation-based diagnostics.

Prediction-based diagnostic was performed by comparing model-derived predictions (PRED, IPRED) with observed ADM concentrations in the external dataset. Mean prediction error (MPE, Equation (1)) and root mean square error (RMSE, Equation (2)) were calculated for bias and precision, respectively:

(1) $$MPE=\frac{\sum \hat{Y}-Y}{n}$$

(2) $$RMSE=\sqrt{\frac{\sum {\left(\hat{Y}-Y\right)}^{2}}{n}}$$

in which $\hat{Y}$ represents the predicted ADM concentration, Y represents the observed ADM concentration, and *n* is the number of observations [14]. Moreover, median prediction error (MDPE, %) and median absolute prediction error (MAPE, %) were used for bias and imprecision as primary criteria, respectively. Other metrics, such as the proportions of prediction errors within ±20% (F20) and ±30% (F30), can support interpretation as combined indicators of both accuracy and precision [12,15,16]. Relative prediction errors were expressed as a percentage of the measured value (PE (%)) using Equation (3), while MDPE and MDPE are expressed by Equations (4) and (5).

(3) $$PE(\%)=\frac{\hat{Y}-Y}{Y}\cdot 100$$

(4) $$MDPE(\%)=median(\frac{\hat{Y}-Y}{Y}\cdot 100)$$

(5) $$MDAPE(\%)=median(\frac{\left|\hat{Y}\right.-\left.Y\right|}{Y}\cdot 100)$$

Simulation-based diagnostics were performed using the normalized prediction distribution error (NPDE). NPDE was determined using an add-on R package npde (version 3.5, INSERM, Paris, France). A simulation data file was generated by running 1000 simulations in NONMEM based on the final model parameter estimates. The assumption of a standard normal distribution, N(0, 1), for NPDE was evaluated using a Wilcoxon signed-rank test to assess the deviation of the mean from zero, and a Fisher variance test to compare the variance with one. In addition, a Shapiro–Wilk test was applied to assess normality of the NPDE distribution. A global test, based on the adjusted *p*-values from all three tests, was used to identify the best-performing model. Graphical evaluation included quantile–quantile (QQ) plots and histograms of NPDE generated using the NPDE package in R.

### 2.4. Model Development

In addition to evaluating the predictive performance of previously published models, a novel population pharmacokinetic model was developed. For model development, nonlinear mixed-effects modeling analysis was performed in NONMEM^®^ (version 7.6, Icon Development Solutions, Ellicott City, MD, USA) [13] together with a compiler using Perl-Speaks NONMEM (PsN, version 5.6.0). Additionally, Pirana (version 2.9.4), R program (version 4.4.2) [17] and various packages (including arsenal, ggplot2, dplyr, xpose) in RStudio (version 2025.09.0+387 “Cucumberleaf Sunflower”) were used for processing NONMEM input and output files. According to the results of the external evaluation of previously published ADM models, the best-performing model was identified and implemented in NONMEM using the $PRIOR NWPRI subroutine to incorporate prior information on a subset of model parameters. This approach was applied due to the sparse nature of our TDM dataset (trough concentrations only), thereby enhancing the robustness of the population pharmacokinetic model and integrating existing knowledge on ADM disposition in CD. CL/F was estimated without prior constraints, as the trough-heavy dataset provided sufficient information for its estimation, while allowing unbiased covariate exploration driven by the study data. A sensitivity analysis was performed by testing different combinations and weightings of prior information, ranging from uninformative to strongly informative priors, to identify the optimal balance between literature-derived knowledge and the informativeness of the current dataset [18].

Informative priors were derived for fixed effects and inter-individual variability from the model identified in the external evaluation step [18]. Parameter estimation was performed using Laplace conditional estimation with interaction. The inter-individual variability was described with an exponential model, while residual variability was evaluated using additive, proportional and combination error models. The M5 method was used for handling BLQ, as these accounted for a very small proportion of the dataset. Notably, BLQ observations were predominantly observed in a limited number of patients who tested positive for ADA; therefore, these data were retained in the analysis. In addition, for the subset of ADM concentrations qualitatively reported as exceeding the therapeutic range (>12 mg/L), the M3 method was applied by treating these observations as right-censored data (like handling the upper limit of quantification—ULQ) [19].

After obtaining the base model, the effect of various covariates (age, sex, body weight, lean body weight, body mass index, hemoglobin, hematocrit, erythrocytes, iron, ferritin, ESR, platelets, leukocytes, ALB, CRP, FCP, immunomodulatory therapy, dosing interval (one week or otherwise), regimen phase (induction, or maintenance phase)) on CL/F was assessed using a stepwise covariate modeling approach. The covariate selection procedure consisted of forward inclusion (*p* < 0.05) and backward elimination (*p* < 0.01), based on changes in the objective function value (OFV) [13,20]. For missing continuous covariates, the patient-specific median or the overall population median was imputed, while missing values for categorical covariates were imputed as zero. Linear, log-linear and power model were tested for continuous covariates.

Model adequacy was evaluated using multiple criteria, including successful minimization and completion of the covariance step, acceptable number of significant digits, stability of gradients in the final iteration, absence of substantial shrinkage, precision of parameter estimates, changes in OFV, and visual inspection of diagnostic plots [13,21,22,23,24]. A prediction- and variability-corrected visual predictive check (pvcVPC) involving 1000 simulations was used for final model evaluation [25]. Robustness of parameter estimates was evaluated using a bootstrap with 1000 samples [21,26] and sampling importance resampling (SIR) procedure [27].

## 3. Results

### 3.1. Review of Selected Literature Models

**Figure 1:**
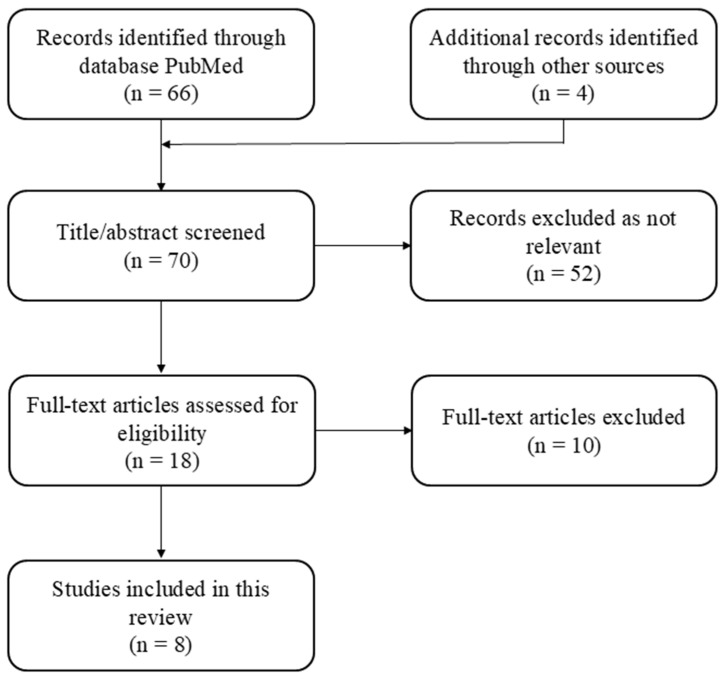
Flow chart of the literature search and study selection process.

A total of 66 records were identified through the PubMed search using the keywords specified in the Methods section. An additional four records were retrieved through an independent literature search. Following screening of titles and abstracts, as well as full-text assessment for eligibility based on the predefined inclusion and exclusion criteria, eight population pharmacokinetic models of ADM were selected (Figure 1).

All selected population models described ADM pharmacokinetics using a one-compartment model with first-order absorption and elimination. Regarding the software used, all models were developed or updated using either NONMEM^®^ or Monolix^®^ program. Specifically, 3 models were developed using NONMEM^®^, 3 using Monolix^®^ software, while the remaining 2 represent adaptations or refinements of previously published population pharmacokinetic models. Further details on the selected studies and models are provided in Table 1.

Typical values for CL/F in the studies ranged from 0.317 to 0.7488 L/day, regardless of the disease type, while typical values of V/F ranged from 4.07 to 13.5 L. The absorption rate constant (Ka) value was usually fixed at 0.15 or 0.2 1/day, or estimated in the range 0.15–0.376 1/day. All models reported that the IIV (percent coefficient of variation) was associated with CL/F, four models reported IIV related to V/F, and only one model estimated IIV for Ka. IOV was accounted by Wright et al. (individuals had up to 4 occasions each around 14 weeks apart) [10] with an effect on the CL/F and V/F, while Spencer et al. accounted IOV only on CL/F value [30]. The residual variability was described with either the proportional model alone, the additive model alone, or a combination of both proportional and additive models.

Identified covariates with a significant influence on CL/F and V/F are presented in Table 1. Berends et al. conducted a retrospective analysis to develop a population pharmacokinetic model of ADM in patients with CD during induction and maintenance treatment. They identified ADA status and dosing regimen (every week or every other week) as significant covariates affecting ADM CL/F [28]. Vande Casteele et al. performed an observational study with rich sampling to develop a population pharmacokinetic model in patients with moderately-to-severely active CD initiating ADM therapy. They described a significant influence of ADA status and lean body weight on CL/F. Since a log-transformed approach was used on both sides, predicted ADM serum concentrations were back-transformed from the natural logarithmic scale prior to the evaluation of prediction metrics [2]. Sánchez-Hernández et al. developed a model in patients with IBD and selected 4 factors with potential clinical relevance for CL/F: body mass index, FCP, unexplained decline in serum ADM concentrations, and pen device formulation (40 mg vs. 80 mg). The external evaluation analysis excluded the last covariate since the 80 mg dosage form was not available in our dataset [7]. Ternant et al. reported a quantitative description of ADM pharmacokinetics in patients with CD and reported a 5.5-fold increase in CL/F in the presence of ADA, with no other significant covariates identified in the model [29]. Wright et al. developed a population pharmacokinetic model that includes ALB level in addition to ADA status [10]. On the other hand, Spencer et al. described the influence of body weight alongside ALB [30]. For models including ADA status, when this covariate was missing in the external evaluation dataset, a negative ADA status was assumed. In contrast, de Klaver et al. [9] and Marquez-Megias et al. [31] adapted or optimized previously published population pharmacokinetic models of ADM. While de Klaver et al. reported significant effects of ADA status and body weight on CL/F [9], Marquez-Megias et al. identified an effect of ALB on CL/F and used ADA status as a fixed parameter [31].

### 3.2. Patients’ Characteristics

Our cohort for external evaluation included data from 195 patients with CD. Baseline characteristics of patients and co-therapy are given in Table 2. A total of 942 ADM trough concentration measurements were obtained during the follow-up period, from which 15 were BLQ (1.6%) and 70 ULQ (7.4%). The mean of the remaining 857 measured concentrations was 9.58 ± 5.69 mg/L. More specifically, average concentration was 9.89 ± 4.90 mg/L for induction phase, and 9.57 ± 5.70 mg/L for maintenance phase. Average number of measured concentrations per patient was 4.46 ± 2.92, ranging from 1 to 14. ADA status was measured on 294 occasions, and it was found positive in only 9 patients (3.06%).

### 3.3. Prediction-Based Diagnostics

The prediction-based diagnostic results are summarized in Table 3. Although none of the models met the combined criteria of MDPE ≤ ±20%, MDAPE ≤ 30%, F20 ≥ 35%, and F30 ≥ 50% (Table 3), the models proposed by Ternant et al. [29] and de Klaver et al. [9] exhibited better characteristics for most metrics compared to other models. Specifically, Ternant et al. demonstrated the most favorable performance, exhibiting the lowest MPE and RMSPE for PRED, as well as superior RMSPE, F20, F30, and MDAPE values for IPRED. De Klaver et al. demonstrated superior F20, F30, and MDAPE values for PRED, as well as the most favorable MDPE for IPRED.

### 3.4. Simulation-Based Diagnostics

**Figure 2:**
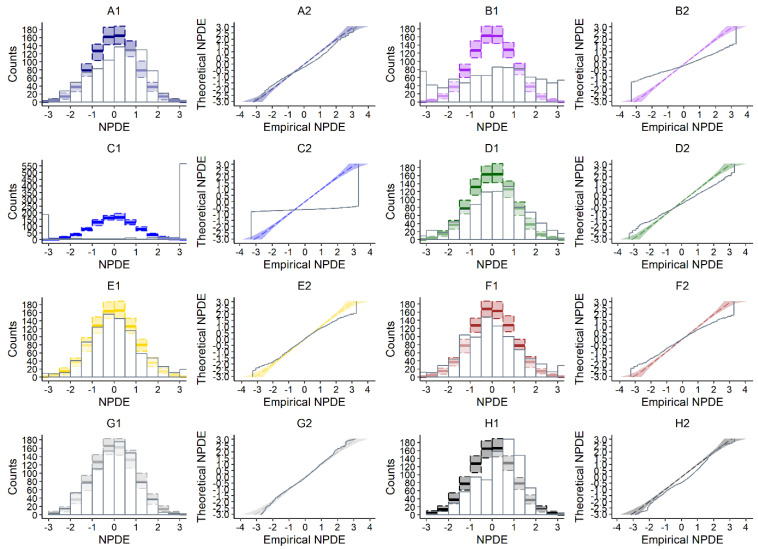
Plots illustrating normalized prediction distribution errors (NPDEs) of the evaluated models; histogram of NPDEs of models (1) and QQ-plot of theoretical NPDE vs. empirical NPDE of models (2): (**A1,A2**) Berends et al. 2018 [28], (**B1,B2**) Vande Casteele et al. 2019 [2], (**C1,C2**) Sánchez-Hernández et al. 2020 [7], (**D1,D2**) Ternant et al. 2015 [29], (**E1,E2**) Wright et al. 2024 [10], (**F1,F2**) Spencer et al. 2024 [30], (**G1,G2**) de Klaver et al. 2023 [9] and (**H1,H2**) Marquez-Megias et al., 2023 [31].

Plots illustrating the NPDE of the evaluated models are shown in Figure 2, while the results of the statistical analysis are summarized in Table S1 (Supplementary File). The statistical evaluation indicated that, for all models, the NPDE deviated from the expected normal distribution N(0, 1).

### 3.5. Population Pharmacokinetic Analysis

Following the superior performance of the model proposed by Ternant et al. [29], it was selected as the base model for further refinement using our clinical dataset. The selected model was a one-compartment model with first-order absorption and linear elimination, parameterized in terms of CL/F, V/F, and Ka, with a combined residual error model. Ka was fixed, and the presence of ADA was included as a categorical covariate on CL/F. During our model development, the effect of this covariate was retained in the model a priori, as positive ADA status is a well-established determinant of ADM CL/F, while its low prevalence in our cohort limited the ability to reliably estimate its effect from the data. Ka was also kept fixed at the original value (0.15 1/day).

Parameter re-evaluation on our dataset, both without prior information and with weakly informative priors on V/F, IIV on V/F, and the ADA effect, resulted in failed minimization due to the sparseness of the data. Given that our real-world dataset consisted exclusively of trough concentrations at steady-state, which are inherently uninformative for Ka and V/F, a full re-estimation of all parameters was numerically unstable. Furthermore, the low prevalence of ADA in our cohort limited the statistical power to independently re-estimate the magnitude of the immunogenicity effect, thereby justifying the use of informative priors to ensure model stability.

Testing various combinations of uninformative and informative priors for the selected parameters revealed that incorporating informative priors for V/F, IIV of V/F, and the ADA effect provided optimal balance of model stability and parameter precision. Notably, both the typical value and IIV of CL/F remained robust across all tested scenarios, with typical values ranging from 0.357 to 0.379 L/day (RSE 3.5–3.9%) and IIV from 37 to 40.5% (RSE 7.4–8.2%). This consistency confirms that the CL/F estimation was predominantly driven by the study data rather than prior specifications.

The stepwise covariate modeling identified CRP and dosing regimen as the only statistically significant covariates for CL/F (*p* < 0.01) in addition to the a priori included effect of ADA. Given the skewed distribution of CRP and the linear trend observed in the empirical Bayes estimates (EBE)-based scatter plots of CL/F vs. ln(CRP), a log-linear relationship was incorporated into the model. This specification resulted in a more pronounced decrease in the OFV and improved parameter precision (lower RSE%) compared to a simple linear model. The inclusion of ln(CRP) effectively eliminated the trend in the diagnostic EBE plots (Figure S1, Supplementary File). Furthermore, CL/F was 21.8% higher in patients receiving weekly administration compared to those on the standard biweekly schedule. Following the inclusion of these covariates, IIV for CL/F was reduced, reaching 33.3% in the final model. Parameter estimates for the base and final models along with results of bootstrap and SIR uncertainty analysis are provided in Table 4. The final parameterization for CL/F is presented in Equation (6). Goodness-of-fit (GOF) diagnostics is presented in Figure S2, Supplementary File.

(6) $$CL/F=0.334\cdot \left(1+6.9\cdot ADA\right)\cdot \left(1+0.075\cdot ln\left(CRP\right)\right)\cdot (1+0.218\cdot REGIMEN)$$

A pvcVPC involving 1000 simulations showed good agreement between observed data and model predictions (Figure S3, Supplementary File).

## 4. Discussion

Individualization of biologic exposure remains challenging due to pronounced pharmacokinetic variability; however, this variability can be more effectively characterized and predicted using population pharmacokinetic models as a priori information. In this study, eight population pharmacokinetic models of ADM in adults were externally evaluated to assess their predictive performance and suitability for dosing regimen optimization. Although two previous studies evaluated the published models using an external dataset [1,28], our analysis includes more recent publications, and is further complemented by the development of a new model informed by the evaluation results. Importantly, external validation across different patient populations is essential to confirm model reproducibility and generalizability. Hence, model evaluation was performed using an external dataset with sparse TDM sampling, applying both prediction-based and simulation-based diagnostic approaches.

Previous population analyses characterized the pharmacokinetics of ADM by a one-compartment model with first-order absorption and elimination (Table 1). Typical values of CL/F in the studies ranged from 0.317 to 0.7488 L/day, indicating high variability. According to the selected models, the covariates that significantly influenced CL/F were ADA status, body size descriptors (body weight, lean body weight or body mass index), FCP, ALB, dosing regimen and pen device, as well as unexplained decline in serum ADM concentrations. However, a substantial proportion of the variability in CL/F (Table 1) remains unexplained, indicating that key determinants of ADM pharmacokinetics have yet to be fully elucidated.

In our external evaluation analysis (Table 3), none of the models satisfied all predefined criteria (MDPE% within ±20%, MDAPE% ≤ 30%, F20 ≥ 35%, and F30 ≥ 50%) [12,15]. In terms of numerical diagnostics, MDPE were used for bias and MDAPE for imprecision as primary criteria, while other metrics can support interpretation. We choose to report several metrics because they do not always lead to the same conclusion [12]. For population-level metrics, MDPE values were within ±20% in four studies [2,9,28,29], whereas MDAPE values were not below 30% in any study. As a combination predictor of both accuracy and precision, F20 and F30 were no more than 35% and 50% in all studies, respectively (Table 3). For individual-level metrics, MDPE values were within ±20% in all studies, with the best result observed for de Klaver et al. [9], followed by Ternant et al. [29]. However, based on MPE results, only two models had confidence intervals (CIs) that included zero [10,30]. Given the pronounced skewness and variability observed in the MPE distributions across models, MDPE likely provides a more robust and reliable measure of the overall trend. MDAPE values were below 30% in most studies [2,9,10,28,29,30,31], while best result was achieved by Ternant et al. [29]. Similarly, F20 and F30 were more than 35% and 50% in most studies, respectively (Table 3), while the best result was achieved by Ternant et al. [29].

Simulation-based diagnostics (Figure 2, Table S1, Supplementary File) do not support the use of evaluated models for population-level simulation tasks [12], including hypothetical “what if” scenarios [32]. Moreover, all models exhibited bias in population predictions, indicating limited utility for a priori dose adjustments based solely on typical population parameters and identified covariates. Among the evaluated models, those developed by Ternant et al. [29], de Klaver et al. [9] and Berends et al. [28] demonstrated superior performance in terms of population-level metrics, although none met all predefined criteria. Regarding NPDE, the model by de Klaver et al. [9] showed better performance among the other models (Table S1, Supplementary File); however, predefined criteria were still not satisfied. In contrast, prediction-based diagnostics at the individual level were generally acceptable across most metrics, with the model by Ternant et al. demonstrating superior performance compared to other models. Notably, a previous external validation study, similarly identified the models by Ternant et al. [29] and Berends et al. [28] as the best-performing models for the studied population, showing lower bias and improved NPDE performance [1]. Given the poor performance of population-level predictions but acceptable individual-level predictions in our analysis, parameter re-estimation was considered. However, due to the sparsity of the evaluation dataset, as expected for TDM data, direct parameter estimation was not possible. Accordingly, the $PRIOR subroutine implemented in NONMEM^®^ was utilized to balance the information from the model with the observed data [12]. Finally, considering that the model by Klaver et al. represents an updated version of a previously published model (Table 2), and that the model by Ternant et al. demonstrated superior individual predictive performance, the latter was selected as the preferred candidate for subsequent analyses. It should be acknowledged that a limitation of IPRED-based metrics is that predictions are estimated using all available observations per individual and therefore do not reflect the ability to predict future concentrations. Hence, for TDM applications of a specific model, Bayesian forecasting provides a more realistic assessment. Nevertheless, as discussed above, we opted to develop a new population pharmacokinetic model using the $PRIOR subroutine. Informative priors were applied using the NONMEM $PRIOR approach after models without priors failed to converge or yielded imprecise parameter estimates, and prior weights were selected based on the reported precision of published parameter estimates with accompanying sensitivity analyses to ensure robustness.

Consequently, a novel population pharmacokinetic model for ADM was developed, where priors for V/F, IIV of V/F, and the ADA effect on CL/F were informed by the parameters established by Ternant et al. [29]. The typical CL/F value estimated in our study aligns with previously reported ranges for IBD patients (Table 1). As shown in previous studies, ADA status is the most common covariate for ADM. In line with this, and given its established role in ADM disposition, ADA status was incorporated as an a priori covariate in our model. This approach ensured that the known impact of immunogenicity on accelerated CL/F was accounted for, regardless of the statistical power of our specific dataset to detect it independently. While the inclusion of ADA status was supported by extensive prior evidence and biological plausibility, it should be acknowledged that this approach partially reduces the independence of the developed model from previously published knowledge. Nevertheless, we considered its inclusion justified to enhance model robustness and clinical relevance. Based on systematic review, incidence of ADA against ADM ranges from 0.3 to 35% in CD patients [33], while pooled results from meta-analysis indicate ADA rate of 7.5% for ADM [34]. These findings indicate that ADA development is not uncommon in clinical practice, although its reported frequency is highly variable across studies. The development of ADA is strongly influenced by the absence of concomitant immunomodulator therapy, low ADM exposure, and higher disease activity, highlighting the close interplay between immunogenicity, drug pharmacokinetics, and the underlying inflammatory burden in patients with IBD. In clinical practice, inflammatory biomarkers such as CRP, FCP, and ferritin serve not only as indicators of disease activity but also as indirect predictors of pharmacokinetic variability and ADA development. Our analysis quantified these relationships, identifying CRP (modeled via a log-linear relationship) and dosing regimen, alongside the a priori inclusion of ADA status, as the significant covariates for ADM.

Inflammatory biomarkers such as ALB and FCP were also identified in several selected studies (Table 1), but not CRP. Although the study by Ponce-Bobadilla et al. did not provide sufficient information for code reconstruction and external evaluation, its findings should be acknowledged, as the authors described the influence of several covariates on CL/F, including ALB, CRP, FCP, indication, body weight, and described ADA-dependent parameters [35]. Interestingly, CRP was not commonly identified in IBD patients as a covariate, except in this study, but has been more frequently observed in other patient populations [36]. Nevertheless, as previously mentioned, the complex interplay between inflammatory burden and pharmacokinetics should be considered. One study in patients with rheumatoid arthritis characterized the relationship between ADM concentrations and CRP using an indirect response model with the inhibition of CRP input [37]. However, in our study, the sparsity of the TDM dataset, characterized by extended intervals of several months between observations, precluded the development of a similar indirect response structure. Although findings derived from patients with rheumatoid arthritis may not be directly transferable to patients with CD [2], they support the concept that inflammatory status is not only a consequence of ADM therapy, but may also represent a significant source of pharmacokinetic variability in IBD patients. Accordingly, our finding suggests that elevated CRP levels may reflect a higher inflammatory burden, potentially contributing to ADM elimination. Conversely, greater disease severity may itself be associated with enhanced drug elimination, indicating a bidirectional relationship. This is particularly relevant as exposure-response analyses often assume that exposure causally drives response. However, this assumption can be violated when the response variable, in this case, disease activity, influences the pharmacokinetic properties of the drug, potentially leading to a biased and over-estimated relationship [38]. A similar interpretation may apply to the observed effect of dosing interval. In our model, more frequent dosing was associated with higher CL/F; however, this finding is likely confounded by disease severity rather than reflecting a causal relationship. So, the variable regimen (standard vs. intensified dosing) was included as a clinical covariate reflecting real-world dose adjustment in response to disease activity and/or inadequate exposure. It should be interpreted as a marker of treatment adaptation rather than a direct mechanistic determinant of CL/F. Consistently, Berends et al. reported higher CL/F in patients receiving weekly ADM administration compared with those treated every other week [28].

Our study has several limitations. The literature search was limited to PubMed, which may have resulted in omission of relevant studies indexed in other databases such as Embase or Scopus. Due to the nature of routine TDM-based data collection, certain covariates, particularly for ALB and FCP, were not available at all observation occasions. Although missing values were handled using median-based imputation, this may have reduced the precision and robustness of covariate effect estimation and limited the ability to detect weaker exposure–covariate relationships. This reflects real-world clinical practice rather than systematic data absence, as these biomarkers were not consistently measured at every visit. Consequently, missingness occurred at the level of monitoring occasions rather than patients, which was addressed using pragmatic imputation approaches as described in the Methods section. Also, only trough concentrations of ADM were available for analysis, which limits the ability to reliably estimate absorption-related parameters, particularly Ka, due to insufficient information on the absorption phase, thereby affecting parameter identifiability. ADA were not assessed systematically in all patients but were measured primarily in cases of low ADM concentrations and/or suspected treatment failure. Consequently, the reported prevalence of ADA positivity may not reflect the true prevalence in the overall cohort, particularly as sensitive assays were used, and hence, the results should be interpreted with caution. Furthermore, the limited number of ADA-positive samples in our cohort may have restricted the statistical power to independently characterize the magnitude of the immunogenicity effect, further justifying the use of a prior-knowledge-based approach for this covariate. Moreover, as our study was based on routine clinical monitoring, the majority of ADM concentrations were measured during the maintenance phase of therapy. Consequently, the pharmacokinetic observations may not be fully generalizable to the induction phase, where rapid fluctuations in inflammatory burden and drug disposition are more pronounced. These limitations may have affected both the generalizability and predictive performance of the results. Hence, future studies based on more comprehensive datasets and prospective designs are warranted to validate existing and the newly developed model presented in this manuscript. Nevertheless, this analysis reflects real-world clinical practice. Furthermore, the factors identified in the developed model as being associated with increased CL/F may aid in identifying individuals at higher risk of therapeutic failure or loss of response.

## 5. Conclusions

We evaluated eight published ADM population pharmacokinetic models in patients with CD using an independent dataset from our center. Their external predictive performance was suboptimal in prediction-based diagnostics, indicating that a priori predictions remain challenging. In addition, the models were not suitable for simulation-based applications, including “what-if” scenarios. However, individual-level prediction-based diagnostics were generally acceptable across most metrics, with the model developed by Ternant et al. demonstrating superior performance compared to other evaluated models. Accordingly, this model served as a source for the prior approach in developing a novel population pharmacokinetic model of ADM. In addition to ADA, the model identified inflammatory burden, reflected by CRP, and dosing interval as significant contributors to variability in ADM CL/F. This model may support Bayesian individualization within an MIPD framework in clinical practice; however, prospective clinical validation is still required before clinical use.

## Figures and Tables

**Table 1:** Summary of selected population pharmacokinetic models of adalimumab (ADM) in patients with inflammatory bowel disease (IBD) and identified covariates of variability.

Reference First Author, Year	Population Number of Subjects (Disease Type); Age	Software/Model	Typical Value of Pharmacokinetic Parameters, IIV, IOV and RV	Covariates Significantly Influenced CL/F or V/F
Developed population pharmacokinetic models using NONMEM software				
Berends, 2018 [28]	96 patients (CD); age median (IQR): 38 (32–44) years	NONMEM/ 1-COMP model with first-order absorption and elimination	CL/F: 0.32 L/day V/F: 4.07 L Ka: 0.2 1/day fixed IIV CL/F: 49.1% Additive error: 1.02 mg/L Proportional error: 0.30	CL/F: ADA status, dosing (every week, or every other week)
Vande Casteele, 2019 [2]	28 patients (CD); age median (IQR): 37 (30–49) years	NONMEM/ 1-COMP model with first-order absorption and elimination	CL/F: 0.33 L/day V/F: 7.8 L Ka: 0.343 1/day IIV CL/F: 32.6% IIV V/F: 35.6% IIV Ka: 103.9% corr Ka-V/F: 0.402 corr Ka-CL/F: −0.576 corr V/F-CL/F: −0.340 Proportional error [additive on the Ln scale]: −16.6%	CL/F: ADA status, lean BW
Sánchez-Hernández, 2020 [7]	104 patients (IBD); age median (IQR): 43 (32–56) years	NONMEM/ 1-COMP model with first-order absorption and elimination	CL/F: 0.3768 L/day V/F: 11.2 L Ka: 0.15 1/day fixed IIV CL/F: 23.2% Proportional error: 21.7%	CL/F: BMI, FCP, unexplained decline in serum ADM concentrations, pen (40 mg or 80 mg pen device)
Developed population pharmacokinetic models using Monolix software				
Ternant, 2015 [29]	65 patients (CD); age median (range): 37 (17–61) years	MONOLIX/ 1-COMP model with first-order absorption and elimination	CL/F: 0.42 L/day V/F: 13.5 L Ka: 0.15 1/day IIV CL/F: 65% IIV V/F: 48% Additive error: 1.8 mg/L Proportional error: 0.15	CL/F: ADA status
Wright, 2024 [10]	53 patients (CD); age median (IQR): 34 (26–44) years	MONOLIX/ 1-COMP model with first-order absorption and elimination	CL/F: 0.317 L/day V/F: 8.9 L Ka: 0.2 1/day fixed IIV(Ω) CL/F: 0.50 IOV CL/F: 0.32 IOV V/F: 0.71 Additive error: 3.24 µg/mL	CL/F: ADA status, ALB
Spencer, 2024 [30]	113 patients (CD)	MONOLIX/ 1-COMP model with first-order absorption and elimination	CL/F: 0.326 L/day V/F: 6.28 L Ka: 0.2 1/day fixed IIV(Ω) CL/F: 0.414 IOV CL/F: 0.296 Additive error: 2.98 µg/mL	CL/F: BW, ALB
Adapted/optimized previous population pharmacokinetic model				
de Klaver, 2023 [9]	36 patients (14 IBD + 22 rheumatology patients); age median (range): 51.5 (42–58) years	NONMEM/ 1-COMP model with first-order absorption and elimination	CL/F: 0.319 L/day/70 kg V/F: 9.83 L/70 kg Ka: 0.376 1/day IIV CL/F: 46.4% IIV V/F: 54.9% Additive error: 0.617 mg/L Proportional error: 14.5%	CL/F: ADA status, BW V/F: BW
Marquez-Megias, 2023 [31]	54 patients (IBD); age median (range): 43.5 (11–89) years	MONOLIX/ 1-COMP model with first-order absorption and elimination	CL/F: 0.7488 L/day V/F: 7.76 L Ka: 0.15 1/day fixed IIV CL/F: 66.7% IIV V/F: 47.7% Proportional error: 0.547	CL/F: ADA status fixed, ALB

ALB—albumin; ADA—anti-drug antibody; ADM—adalimumab; BMI—body mass index; BW—body weight; CD—Crohn’s disease; CL/F—apparent clearance; COMP—compartment; corr—correlation coefficient; FCP—fecal calprotectin; IBD—inflammatory bowel disease; IIV—inter-individual variability; IOV—interoccasional variability; IQR—interquartile range; Ka—absorption rate constant; RV—residual variability; V/F—apparent volume of distribution.

**Table 2:** Baseline patients’ and co-therapy characteristics.

Characteristic (Units)	Values
Age (years)	
Mean (SD)	38.05 (11.83)
Range	17.00–67.00
Sex	
Male	96 (49.2%)
Female	99 (50.8%)
Body weight (kg)	
N-Miss	19
Mean (SD)	69.44 (13.47)
Range	38.00–105.00
Hemoglobin (g/L)	
N-Miss	106
Mean (SD)	132.37 (19.22)
Range	82.00–165.00
Hematocrit (L/L)	
N-Miss	92
Mean (SD)	0.40 (0.05)
Range	0.25–0.51
Erythrocytes (10^12^/L)	
N-Miss	91
Mean (SD)	4.55 (0.54)
Range	2.90–5.83
Erythrocyte sedimentation rate (mm/h)	
N-Miss	157
Mean (SD)	18.87 (13.59)
Range	2.00–60.00
Iron (µmol/L)	
N-Miss	114
Mean (SD)	12.65 (8.09)
Range	1.50–43.20
C-reactive protein (mg/L)	
N-Miss	57
Mean (SD)	8.46 (13.47)
Range	0.00–72.70
Albumin (g/L)	
N-Miss	180
Mean (SD)	39.27 (5.12)
Range	27.00–46.00
Platelets (10^9^/L)	
N-Miss	110
Mean (SD)	300.59 (92.44)
Range	99.00–601.00
Leukocytes (10^9^/L)	
N-Miss	107
Mean (SD)	7.73 (3.05)
Range	2.80–20.00
Ferritin (ng/mL)	
N-Miss	150
Mean (SD)	80.73 (73.93)
Range	2.20–348.00
Fecal calprotectin (µg/g)	
N-Miss	156
Mean (SD)	806.02 (810.12)
Range	18.00–4213.00
Azathioprine	
No	77 (39.5%)
Yes	118 (60.5%)
Prior anti-TNFα therapy	
No	129 (66.2%)
Yes	66 (33.8%)

Miss—missing data; N—number; SD—standard deviation.

**Table 3:** Comparison of population (PRED) and individual (IPRED) predictions to observed adalimumab (ADM) concentrations for the evaluated models.

Reference (First Author, Year)/ Parameter	MPE (mg/L) 95% CI	MDPE (%)	RMSPE (mg/L)	F20 (%)	F30 (%)	MDAPE (%)	MPE (mg/L) 95% CI	MDPE (%)	RMSPE (mg/L)	**F20** **(%)**	**F30** **(%)**	**MDAPE (%)**
**Predictions**	**PRED**						**IPRED**					
Berends, 2018 [28]	−0.971 −1.34–−0.60	−11.33	5.65	22.52	35.36	40.62	0.477 0.191–0.763	2.24	4.30	39.67	58.23	25.37
Vande Casteele, 2019 [2]	1.029 0.497–1.562	2.07	8.02	19.02	30.34	49.2	−0.766 −1.02–−0.511	−5.07	3.88	40.96	56.94	24.89
Sánchez-Hernández, 2020 [7]	−8.67 −9.04–−8.29	−91.12	10.28	1.17	2.33	91.17	−0.989 −1.273–−0.704	−12.63	4.36	31.27	47.84	31.45
Ternant, 2015 [29]	−0.757 −1.132–−0.382	−8.51	5.65	25.79	37.22	40.09	0.330 0.076–0.584	1.99	3.81	43.52	60.68	24.71
Wright, 2024 [10]	3.80 3.37–4.23	40.20	7.45	24.15	35.94	45.40	0.084 −0.173–0.341	2.57	3.84	41.31	56.13	26.40
Spencer, 2023 [30]	5.81 5.22–6.40	48.11	10.52	21.94	31.97	55.02	0.036 −0.230–0.302	2.43	3.97	39.21	54.49	27.07
de Klaver, 2023 [9]	1.79 1.38–2.20	17.73	6.38	25.9	39.56	39.06	0.347 0.0629–0.631	−0.51	4.25	38.97	58.23	25.89
Marquez-Megias, 2023 [31]	−3.31 −3.68–−2.95	−33.43	6.34	15.17	25.67	51.51	−0.939 −1.217–−0.662	−13.70	4.25	30.81	50.88	29.43

CI—confidence interval; F20—percentage of PE% within ±20%; F30—percentage of PE% within ±30%; MDAPE—median absolute prediction error; MDPE—median prediction error; MPE—mean prediction error; RMSPE—root mean square prediction error.

**Table 4:** Parameter estimates for the base and final population pharmacokinetic model of adalimumab (ADM).

Parameter (Units)	Base Model	Final Model		
	Original data	Original data	Bootstrap	Sampling Importance Resampling (SIR)
	Estimate (%RSE)	Estimate (%RSE)	Median (95% CI)	Median (95% CI)
V/F (L)	13.9 (9.4)	13.5 (6.6)	13.6 (12.2–14.5)	13.5 (11.5–15.4)
Ka (day^−1^)	0.15 (fix)	0.15 (fix)	0.15	0.15
CL/F (L/day)	0.376 (3.5)	0.334 (3.4)	0.333 (0.313–0.361)	0.335 (0.314–0.360)
ADA on CL/F	6.94 (31.6)	6.90 (28.4)	6.97 (5.61–8.62)	6.98 (3.45–11.15)
ln(CRP) on CL/F	/	0.075 (20.1)	0.076 (0.042–0.112)	0.073 (0.044–0.102)
Dosing regimen on CL/F	/	0.218 (20.7)	0.217 (0.128–0.317)	0.217 (0.129–0.301)
IIV_V_ (%)	49.9 (21.3)	53.8 (10)	51.5 (46.5–70.11)	53.5 (41.7–64.7)
IIV_CL_ (%)	40.5 (8.1)	33.3(8.3)	33.2 (26.1–39.7)	33.5 (27.9–38.5)
Additive residual error (mg/L)	2.88 (9.7)	2.71 (7.6)	2.65 (1.34–3.51)	2.71 (2.27–3.17)
Proportional residual error	0.323 (9.0)	0.337 (6.9)	0.342 (0.248–0.425)	0.341 (0.289–0.390)

ADA—anti-drug antibody; CI—confidence interval; CL/F—apparent clearance; CRP—C-reactive protein; IIV—inter-individual variability; Ka—absorption rate constant; RSE—relative standard error; V/F—apparent volume of distribution.

## Data Availability

The original contributions presented in this study are included in the article/Supplementary Material. Further inquiries can be directed to the corresponding author.

## Supplementary Materials

The following supporting information can be downloaded at https://www.mdpi.com/article/10.3390/pharmaceutics18070788/s1, Table S1: Statistical tests for evaluating the normality of the normalized prediction distribution errors (NPDEs); Figure S1. Impact of C-reactive protein (CRP) on adalimumab clearance (CL/F); Figure S2. Goodness-of-fit diagnostics for the final population pharmacokinetic model of adalimumab. Observations (DV) versus: (A) population predictions (PRED), (B) individual predictions (IPRED); Conditional weighted residuals (CWRES) versus: (C) time, (D) PRED. The locally regression line (red lines). (A,B) the line of unity (blue solid lines), and (C,D) y = 0 (blue solid lines); Figure S3: Prediction- and variability-corrected visual predictive check (pvcVPC) for adalimumab (ADM). Solid lines represent the median, 5th and 95th percentiles of the original observations, while the shaded area represent confidence intervals of simulated data.

## References

[1] Marquez-Megias S., Ramon-Lopez A., Mas-Serrano P., Diaz-Gonzalez M., Candela-Boix M.R., Nalda-Molina R. (2021). Evaluation of the Predictive Performance of Population Pharmacokinetic Models of Adalimumab in Patients with Inflammatory Bowel Disease. Pharmaceutics.

[2] Vande Casteele N., Baert F., Bian S., Dreesen E., Compernolle G., Van Assche G., Ferrante M., Vermeire S., Gils A. (2019). Subcutaneous Absorption Contributes to Observed Interindividual Variability in Adalimumab Serum Concentrations in Crohn’s Disease: A Prospective Multicentre Study. J. Crohns Colitis.

[3] Humira S.P.C. Summary of Product Characteristics for Humira 40 mg Solution for Injection in Pre-Filled Pen. https://www.medicines.org.uk/emc/product/7986/smpc#gref.

[4] Billioud V., Sandborn W.J., Peyrin-Biroulet L. (2011). Loss of response and need for adalimumab dose intensification in Crohn’s disease: A systematic review. Am. J. Gastroenterol..

[5] Srinivasan A., Gilmore R., van Langenberg D., De Cruz P. (2022). Systematic review and meta-analysis: Evaluating response to empiric anti-TNF dose intensification for secondary loss of response in Crohn’s disease. Ther. Adv. Gastroenterol..

[6] Vande Casteele N., Gils A. (2015). Pharmacokinetics of anti-TNF monoclonal antibodies in inflammatory bowel disease: Adding value to current practice. J. Clin. Pharmacol..

[7] Sanchez-Hernandez J.G., Perez-Blanco J.S., Rebollo N., Munoz F., Prieto V., Calvo M.V. (2020). Biomarkers of disease activity and other factors as predictors of adalimumab pharmacokinetics in inflammatory bowel disease. Eur. J. Pharm. Sci..

[8] Deyhim T., Cheifetz A.S., Papamichael K. (2023). Drug Clearance in Patients with Inflammatory Bowel Disease Treated with Biologics. J. Clin. Med..

[9] de Klaver P.A.G., Keizer R.J., Ter Heine R., Smits L., Boekema P.J., Kuntzel I., Schaap T., de Vries A., Bloem K., Rispens T. (2023). Early At-Home Measurement of Adalimumab Concentrations to Guide Anti-TNF Precision Dosing: A Pilot Study. Eur. J. Drug Metab. Pharmacokinet..

[10] Wright E.K., Chaparro M., Gionchetti P., Hamilton A.L., Schulberg J., Gisbert J.P., Chiara Valerii M., Rizzello F., De Cruz P., Panetta J.C. (2024). Adalimumab Clearance, Rather Than Trough Level, May Have Greatest Relevance to Crohn’s Disease Therapeutic Outcomes Assessed Clinically and Endoscopically. J. Crohns Colitis.

[11] El Hassani M., Marsot A. (2023). External Evaluation of Population Pharmacokinetic Models for Precision Dosing: Current State and Knowledge Gaps. Clin. Pharmacokinet..

[12] El Hassani M., Marsot A. (2026). Guidance for External Evaluation and Selection of Population Pharmacokinetic Models for Precision Dosing. Clin. Pharmacokinet..

[13] Beal S.L., Sheiner L.B., Boeckmann A.J., Bauer R.J. NONMEM Users Guides.

[14] Sheiner L.B., Beal S.L. (1981). Some suggestions for measuring predictive performance. J. Pharmacokinet. Biopharm..

[15] Chen S., Huang L., Huang W., Zheng Y., Shen L., Liu M., Chen W., Wu X. (2024). External Evaluation of Population Pharmacokinetic Models for High-Dose Methotrexate in Adult Patients with Hematological Tumors. J. Clin. Pharmacol..

[16] Zhang H.X., Sheng C.C., Liu L.S., Luo B., Fu Q., Zhao Q., Li J., Liu Y.F., Deng R.H., Jiao Z. (2019). Systematic external evaluation of published population pharmacokinetic models of mycophenolate mofetil in adult kidney transplant recipients co-administered with tacrolimus. Br. J. Clin. Pharmacol..

[17] R Core Team (2026). R: A Language and Environment for Statistical Computing.

[18] Chan Kwong A.H.P., Calvier E.A.M., Fabre D., Gattacceca F., Khier S. (2020). Prior information for population pharmacokinetic and pharmacokinetic/pharmacodynamic analysis: Overview and guidance with a focus on the NONMEM PRIOR subroutine. J. Pharmacokinet. Pharmacodyn..

[19] Irby D.J., Ibrahim M.E., Dauki A.M., Badawi M.A., Illamola S.M., Chen M., Wang Y., Liu X., Phelps M.A., Mould D.R. (2021). Approaches to handling missing or “problematic” pharmacology data: Pharmacokinetics. CPT Pharmacomet. Syst. Pharmacol..

[20] Jonsson E.N., Karlsson M.O. (1998). Automated covariate model building within NONMEM. Pharm. Res..

[21] Mould D.R., Upton R.N. (2013). Basic concepts in population modeling, simulation, and model-based drug development-part 2: Introduction to pharmacokinetic modeling methods. CPT Pharmacomet. Syst. Pharmacol..

[22] Karlsson M.O., Savic R.M. (2007). Diagnosing model diagnostics. Clin. Pharmacol. Ther..

[23] Savic R.M., Karlsson M.O. (2009). Importance of shrinkage in empirical bayes estimates for diagnostics: Problems and solutions. AAPS J..

[24] Hooker A.C., Staatz C.E., Karlsson M.O. (2007). Conditional weighted residuals (CWRES): A model diagnostic for the FOCE method. Pharm. Res..

[25] Bergstrand M., Hooker A.C., Wallin J.E., Karlsson M.O. (2011). Prediction-corrected visual predictive checks for diagnosing nonlinear mixed-effects models. AAPS J..

[26] Parke J., Holford N.H., Charles B.G. (1999). A procedure for generating bootstrap samples for the validation of nonlinear mixed-effects population models. Comput. Methods Programs Biomed..

[27] Dosne A.G., Bergstrand M., Karlsson M.O. (2017). An automated sampling importance resampling procedure for estimating parameter uncertainty. J. Pharmacokinet. Pharmacodyn..

[28] Berends S.E., Strik A.S., Van Selm J.C., Lowenberg M., Ponsioen C.Y., D’Haens G.R., Mathot R.A. (2018). Explaining Interpatient Variability in Adalimumab Pharmacokinetics in Patients with Crohn’s Disease. Ther. Drug Monit..

[29] Ternant D., Karmiris K., Vermeire S., Desvignes C., Azzopardi N., Bejan-Angoulvant T., van Assche G., Paintaud G. (2015). Pharmacokinetics of adalimumab in Crohn’s disease. Eur. J. Clin. Pharmacol..

[30] Spencer E.A., Dubinsky M.C., Kamm M.A., Chaparro M., Gionchetti P., Rizzello F., Gisbert J.P., Wright E.K., Schulberg J.D., Hamilton A.L. (2024). Poor prognostic factors of pharmacokinetic origin predict outcomes in inflammatory bowel disease patients treated with anti-tumor necrosis factor-alpha. Front Immunol..

[31] Marquez-Megias S., Nalda-Molina R., Mas-Serrano P., Ramon-Lopez A. (2023). Population Pharmacokinetic Model of Adalimumab Based on Prior Information Using Real World Data. Biomedicines.

[32] Cheng Y., Wang C.Y., Li Z.R., Pan Y., Liu M.B., Jiao Z. (2021). Can Population Pharmacokinetics of Antibiotics be Extrapolated? Implications of External Evaluations. Clin. Pharmacokinet..

[33] Vermeire S., Gils A., Accossato P., Lula S., Marren A. (2018). Immunogenicity of biologics in inflammatory bowel disease. Ther. Adv. Gastroenterol..

[34] Bots S.J., Parker C.E., Brandse J.F., Lowenberg M., Feagan B.G., Sandborn W.J., Jairath V., D’Haens G., Vande Casteele N. (2021). Anti-Drug Antibody Formation Against Biologic Agents in Inflammatory Bowel Disease: A Systematic Review and Meta-Analysis. BioDrugs.

[35] Ponce-Bobadilla A.V., Stodtmann S., Chen M.J., Winzenborg I., Mensing S., Blaes J., Haslberger T., Laplanche L., Dreher I., Mostafa N.M. (2023). Assessing the Impact of Immunogenicity and Improving Prediction of Trough Concentrations: Population Pharmacokinetic Modeling of Adalimumab in Patients with Crohn’s Disease and Ulcerative Colitis. Clin. Pharmacokinet..

[36] Tian X., Yu Y., Neeli H., Jappar D. (2025). Impact of Chronic Inflammatory Diseases on Clinical Pharmacokinetics of Antibody-Based Therapeutic Proteins. Clin. Pharmacol. Ther..

[37] Ternant D., Ducourau E., Fuzibet P., Vignault C., Watier H., Lequerre T., Le Loet X., Vittecoq O., Goupille P., Mulleman D. (2015). Pharmacokinetics and concentration-effect relationship of adalimumab in rheumatoid arthritis. Br. J. Clin. Pharmacol..

[38] Karlsson M.O., Brundavanam D. (2026). Addressing Causality and Homogeneity Assumptions in Exposure-Response Analyses. Clin. Pharmacol. Ther..

