# Supplementary material for: Unlocking the Potential of Population Pharmacokinetic Models of Adalimumab in Patients with Crohn’s Disease
Source: Pharmaceutics. 2026 Jun 27;18(7):788. doi: 10.3390/pharmaceutics18070788 (PMC13416018; doi:10.3390/pharmaceutics18070788)
Supplement: Supplementary file 1 [file pharmaceutics-18-00788-s001.zip › pharmaceutics-4376821-supplementary.pdf]

Unlocking the potential of population pharmacokinetic models of adalimumab in patients with Crohn's disease

Marija Jovanović [1,†], Valentina Topić Vučenović [2,†], Maša Roganović [1], Gordana Pavlović [3], Đorđe Kralj [4], Srđan Marković [4,5], Petar Svorcan [4,5] and Katarina Vučićević [1,*]

[1] Department of Pharmacokinetics and Clinical Pharmacy, Faculty of Pharmacy, University of Belgrade, 11221 Belgrade, Serbia
[2] Department of Pharmacokinetics and Clinical Pharmacy, Faculty of Medicine, University of Banja Luka, 78000 Banja Luka, Bosnia and Herzegovina
[3] Internal Medicine Clinic, University Clinical Hospital Center "Dr Dragiša Mišović—Dedinje", 11000 Belgrade, Serbia
[4] Department of Gastroenterology and Hepatology, University Hospital Medical Center "Zvezdara", 11000 Belgrade, Serbia
[5] Faculty of Medicine, University of Belgrade, 11000 Belgrade, Serbia
* Correspondence: katarina.vucicevic@pharmacy.bg.ac.rs
† These authors contributed equally to this work.

Table S1. Statistical tests for evaluating the normality of the normalized prediction distribution errors (NPDEs).

**Reference** (first author, year)**/Test**	**Wilcoxon***	**Fisher***	**Shapiro-Wilk***	**Global***
Berends, 2018	<0.001	<0.001	<0.001	<0.001
Vande Casteele, 2019	0.854	<0.001	<0.001	<0.001
Sánchez-Hernández, 2020	<0.001	<0.001	<0.001	<0.001
Ternant, 2015	<0.001	<0.001	<0.05	<0.001
Wright, 2024	1	<0.001	<0.001	<0.001
Spencer, 2024	1	<0.001	<0.001	<0.001
de Klaver, 2023	1	1	<0.05	<0.05
Marquez-Megias, 2023	<0.001	1	<0.001	<0.001

*data are expressed as *p*-values.

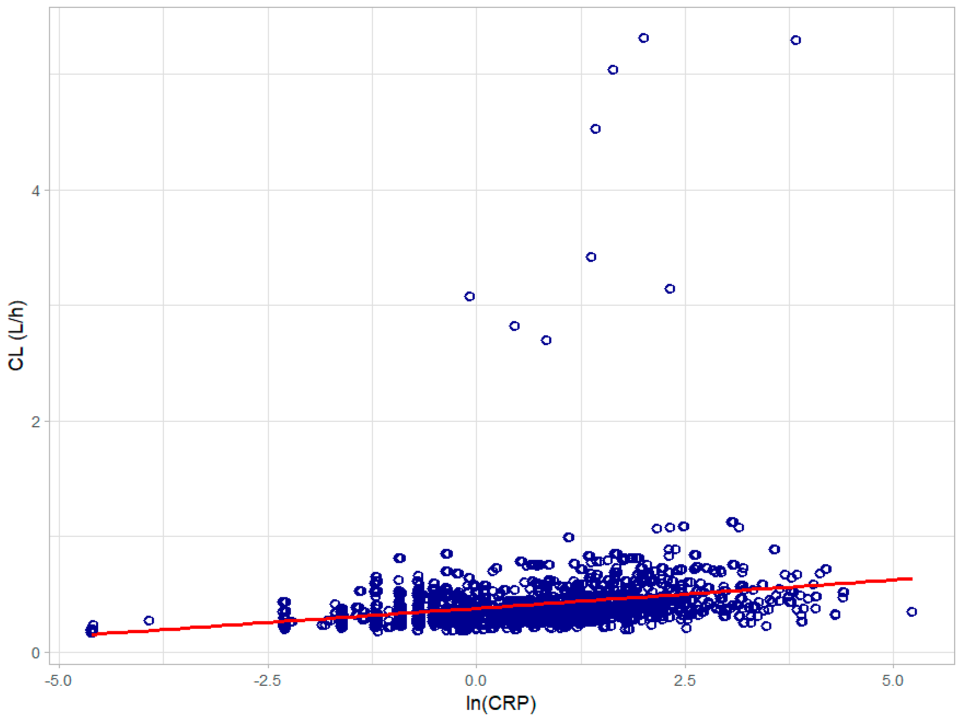

Figure S1. Impact of C-reactive protein (CRP) on adalimumab clearance (CL/F).

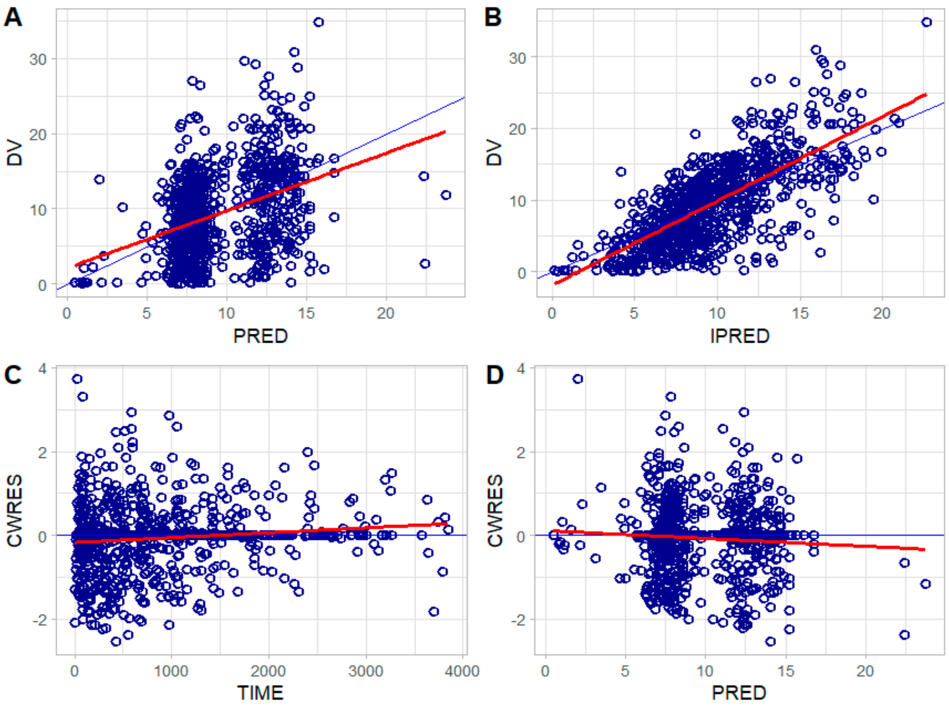

Figure S2. Goodness-of-fit diagnostics for the final population pharmacokinetic model of adalimumab. Observations (DV) versus: (A) population predictions (PRED), (B) individual predictions (IPRED); Conditional weighted residuals (CWRES) versus: (C) time, (D) PRED. The locally regression line (red lines). (A, B) the line of unity (blue solid lines), and (C, D) y = 0 (blue solid lines).

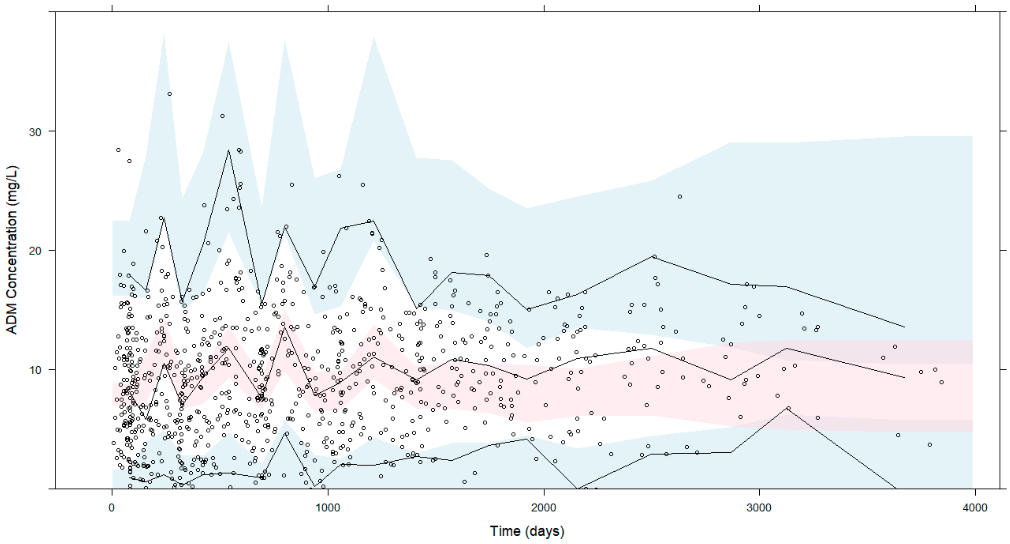

Figure S3. Prediction- and variability-corrected visual predictive check (pvcVPC) for adalimumab (ADM). Solid lines represent the median, 5th and 95th percentiles of the original observations, while the shaded area represent confidence intervals of simulated data.
